# A Community-Based Addiction Rehabilitation Electronic System to Improve Treatment Outcomes in Drug Abusers: Protocol for a Randomized Controlled Trial

**DOI:** 10.3389/fpsyt.2018.00556

**Published:** 2018-11-06

**Authors:** Zhe Wang, Shujuan Chen, Junning Chen, Chunfeng Xu, Zhikang Chen, Wenxu Zhuang, Xu Li, Min Zhao, Jiang Haifeng

**Affiliations:** ^1^Shanghai Mental Health Center, Shanghai Jiao Tong University School of Medicine, Shanghai, China; ^2^Nantong Winner Information Technology Co., Ltd, Nantong, China; ^3^Shanghai Key Laboratory of Psychotic Disorders, Shanghai, China

**Keywords:** mobile health, drug abuse, rehabilitation, community health service, China

## Abstract

**Introduction:** Relapse is very common in drug abusers and contributes to a series of negative consequences. Effective addiction treatment exists but there are some problems in the implementation process. Mobile health (mHealth) offers a potential solution to improving recovery outcome for drug abusers in the community. The research team developed a community-based addiction rehabilitation electronic system (CAREs). The primary aim of this study is to explore whether the integrated rehabilitation based on program CAREs promotes drug abusers to keep abstinence. The secondary aim is to evaluate the impact of CAREs on interaction between drug users and service providers, and on addiction-related physical and social functions.

**Method and analysis:** A randomized controlled trial (RCT) will be conducted. The study is a superiority trial with parallel group design. Seventy drug abusers who are newly ordered to undergo community rehabilitation will be recruited from the community in Shanghai. Participants will be 1:1 randomly assigned to receive integrated community rehabilitation by using CAREs or only receiving routine community rehabilitation for 6 months. Corresponding social workers will provide service and monitor their drug use behavior in accordance with the routine work-flow. Outcomes will be assessed at baseline and in the 6th month. The primary outcome is the performance on illicit drug urine test which will be carried out regularly twice per week during the study period. Secondary study outcomes include longest duration of sustained abstinence, days that participants interact with social workers, and the decrease rate of addiction-related issues severity index. Chi-square tests and ANOVAs will be used to compare characteristics of the members of the two groups. GEE will be used to compare the seven dimensions scores of the ASI between groups.

**Discussion:** The study provides evidence for the feasibility and effectiveness of the “CAREs” system through comparing the results of the intervention group with the control group. This paper describes the design and methodology of the study.

**Ethics and dissemination:** The Ethical Board of SMHC approved the study protocol. All participants will present for the informed consent process. After study completion, the results will be published.

**Trial Registration:** ClinicalTrials.gov NCT03451344, https://clinicaltrials.gov/ct2/show/NCT03451344

## Introduction

Drug abuse is a global public health problem (1). In recent years, the number of individuals who use drugs in China has increased year by year. By the end of 2017, the total number of individuals who use drugs in China has been 2.505 million (2), but the actual number of drug users is much higher than that. In order to control China's increasingly serious drug epidemic phenomenon and related hazards, since the promulgation of the “Anti-drug Law” in 2007, China has established a trinity addiction treatment model with three forms: isolated compulsory treatment, voluntary treatment provided by qualified medical institutions, and treatment of drug addiction in the community (community-based rehabilitation) (3, 4).

Among them, the community-based rehabilitation has become one of the most important measures to treat drug addiction (5). Community-based rehabilitation refers to the efforts of community uniting families, public health and safety agencies, civil affairs departments, and other social resources to help drug abusers to achieve long-term abstinence. Community-based rehabilitation also helps drug abusers to restore physical and mental health, enjoy equal opportunities, and become free from social discrimination. The number of new reported community addiction was 245,000 and the number of community rehabilitation was 59,000 in China in 2016 (2). However, due to the lack of relevant interventions, around 40–60% drug abusers who were ordered to undergo the community-based rehabilitation relapsed, or were incarcerated, were referred to compulsory treatment, or died (6). Therefore, it is a pressing issue to help drug abusers to improve their anti-relapse skills and increase the detoxification rate in the community.

At present, China's community drug treatment and community rehabilitation are still at an initial stage of development and are still immature (7). There are some difficulties and challenges that cannot be ignored. They are mainly manifested in the following aspects: (1) due to limited funding, the community public facilities are inadequate; (2) community workers find it difficult to provide professional and systematic services because of their lack of professional consciousness and professional competence; (3) there is a serious shortage of community staff and a high turnover rate (8). In addition, according to the Anti-drug Law of the People's Republic of China, community drug treatment lasts 3 years, which is a long time for both drug abusers and social workers (3, 7). This brings a further challenge to the community drug treatment and community rehabilitation. Therefore, it is imperative to explore new ways for community drug treatment and community rehabilitation.

Mobile health (mHealth) is a term used for the practice of medicine and public health supported by mobile communication technology (such as PDA, mobile phones, and satellite communications). mHealth applications include the use of mobile devices in collecting community and clinical health data, delivery of healthcare information to practitioners, researchers, and patients, real-time monitoring of patients' vital signs, and direct provision of care. With the popularity of smartphones, the spread of smartphone technologies opens up doors for mHealth projects, particularly for chronic disease management. Since an APP named “WellDoc” approved by the U.S. FDA for the prevention and treatment of diabetes (9), the United States FDA has approved more than 31 mobile health products by 2015. In practice, the main functions of mobile health services include disease and health information, education, disease screening, assessment and monitoring, intervention, and patient self-management, and social support (10). In the study field of drug treatment and rehabilitation, international exploration is conducted through the Internet (11), mobile phone SMS (12, 13) and APPs (14), which offers professional intervention to persons with substance use disorders. At present, the ecological momentary assessment (EMA) is mainly used. The advantage of this technology is to collect real-time data and immediately evaluate and intervene on high-risk factors of patients to prevent relapse (15, 16). The data shows that it has certain effects on providing knowledge of drug addiction and coping skills for high-risk situations for relapse and reducing relapse rates. For cocaine users, using EMA technology to dynamically monitor the relapse factors such as the location and emotional changes of individuals with substance use disorders can significantly improve their self-detection and management capabilities and help them stop using drugs (17). For HIV prevention the participants were sent weekly relevant prevention information in the form of text messages. After 6 months, the results showed that the intervention group had a lower risk of HIV infection than the control group (18). In recent years, the development of mobile health researches has become a hot topic in China, but there is lack of relevant researches in the field of drug addiction.

In 2003, a new model of community-based drug treatment and rehabilitation “Dominated by the Government, Self-operated by the Community and Involved by Society” emerged in Shanghai (19). More than 800 professional social workers in the addiction recovery services are currently providing community services for around 70,000 drug abusers who were ordered to undergo community-based rehabilitation. Although Shanghai has a pioneering attempt in China's community drug treatment and rehabilitation, community service providers and resources remain severely inadequate. There is an urgent need for new means of intervention and technology to improve the efficiency and effectiveness of community drug treatment and rehabilitation. The good news is that China's mobile phone popularity rate has improved significantly and the use of mobile phone APPs has become people's daily behavior (20). Due to the current situation, community-based rehabilitation program in Shanghai exploring the mutual integration of a mHealth rehabilitation model based on the mobile APP is a good opportunity.

The research team developed a community-based addiction rehabilitation electronic system (CAREs) based on a smartphone app, including the functions of education, support, and psychological interventions. CAREs is an electronic information platform for both community drug users and service providers. The goal of the system is to address the social psychological support needs of the persons with substance use disorders in the community, to improve the professionalization and standardization of community rehabilitation, and to further improve the efficiency of community-based drug rehabilitation, and ultimately help the drug users in the community to stay abstinent. In this study, the investigators will use the method of quantitative research to evaluate the effectiveness of using the electronic management system of drug rehabilitation in clinical work. This study is of important social significance to improve the community drug addiction / rehabilitation model in Shanghai and provide important theoretical basis for the future research in the field of substance abuse treatment.

This paper outlines the protocol for a randomized controlled trial (RCT) of the community-based addiction rehabilitation electronic system (CAREs) based on a smartphone application. This interactive system consists of an APP for clients and a webpage for service providers, with the aim of teaching clients craving and emergency coping skills and helping service providers to improve their work efficiency in communities. The primary aim of this study is to explore whether the integrated rehabilitation based on CAREs promotes drug abusers to stay abstinent. The investigators hypothesize that the integrated rehabilitation group will show significant improvement in performance on illicit drug urine tests. The secondary aim is to evaluate the impact of CAREs on the interaction between drug users and service providers, and on addiction-related physical and social functions.

## Methods and analysis

### Trial design

This is a randomized controlled trial (RCT). The study uses parallel group design and is a superiority trial to explore whether the integrated rehabilitation based on CAREs promotes drug abusers to stay abstinent. The study divides participants into integrated and standardized community-based rehabilitation groups in a ratio of 1:1 and has 2 arms: the participants in integrated rehabilitation group will receive regular community-based rehabilitation plus CAREs with their matched social workers, the participants randomized to the standardized community-based rehabilitation group will only receive routine community-based rehabilitation.

### Study setting

The setting for this study is in Shanghai, China. According to the Anti-Drug Law which was issued in 2007, drug abusers discharged from compulsory treatment programs should receive community-based rehabilitation for around 2 years. In Shanghai, community social workers in the addiction recovery services are employed by the government to help drug users and monitor their drug use behavior. There are around 1,000 social workers serving about 70,000 drug users in Shanghai.

### Eligibility criteria

Drug abusers who are newly ordered to undergo community-based rehabilitation will be recruited for the study. Candidates are eligible to participate if they are aged 20–50 years, meeting the DSM-5 (Diagnostic and Statistical Manual of Mental Disorders-V) criteria for substance dependence, and are willing to comply with the relevant requirements of the study including using the mobile app. People who are unable to use mobile phones or have a history of suicidality or a significant cognitive impairment will not be recruited. There is no limitation for gender when recruiting participants.

### Interventions

Participants who are newly enrolled in the standardized community-based rehabilitation will be designated to join the social worker organizations where the residence of the drug users are registered and have to sign a contract with their corresponding social workers. Participants need to visit their corresponding social workers and receive illicit drug test every 2 months. Social workers will help their corresponding clients to apply for social benefits accordingly and provide counseling irregularly if necessary.

The integrated rehabilitation group will receive the standardized community-based rehabilitation described above and simultaneously receive a 6 months integrated rehabilitation based on CAREs. CAREs was developed by the department of addiction research in Shanghai Mental Health Center (SMHC), with functions including education, support, psychological intervention and other functions.

**Figure 1:**
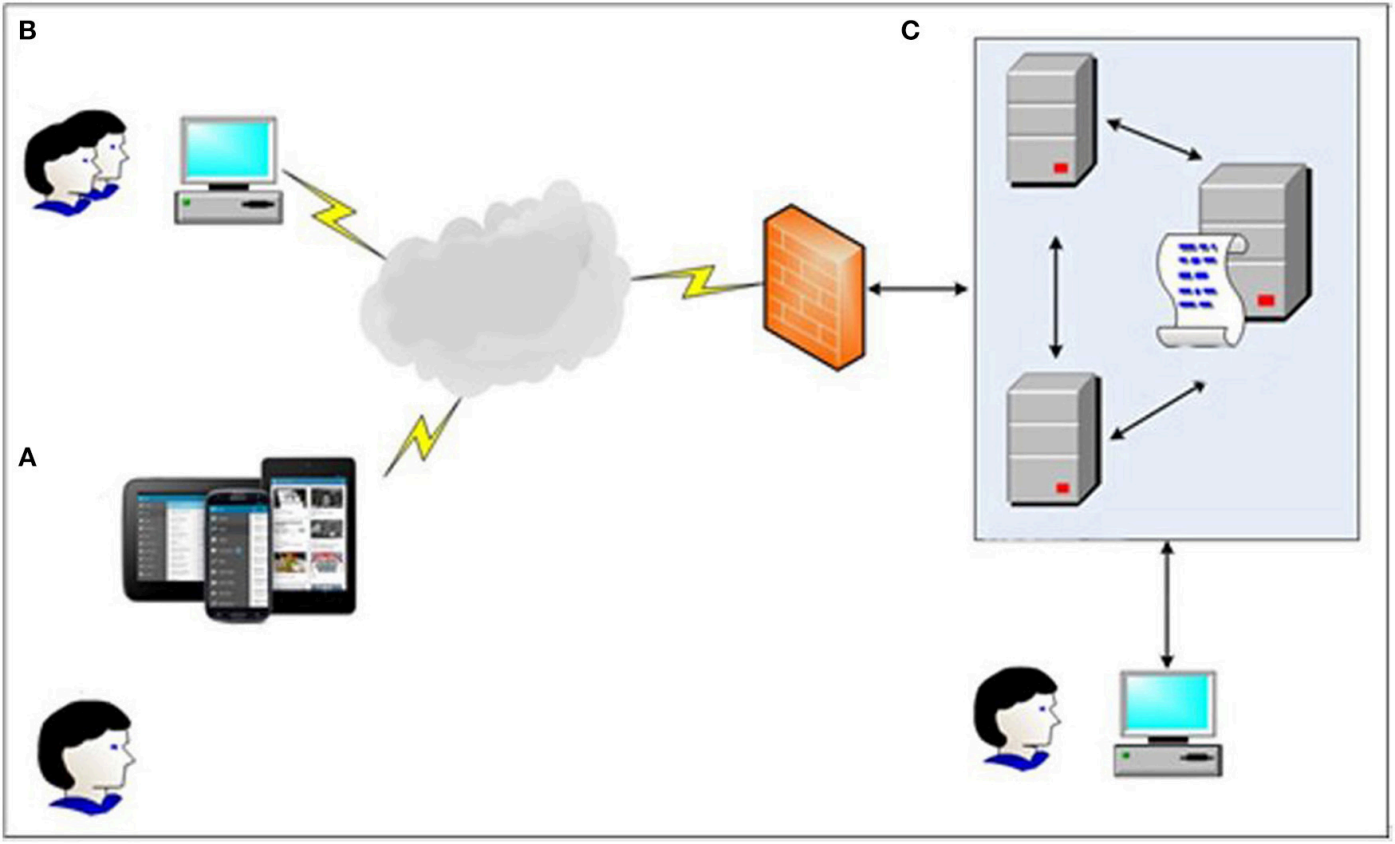
Frame diagram of Community-based Addiction Rehabilitation Electronic System (CAREs): **(A)** Users manage to get immediate support and intervention through CAREs installed in smart phone devices; **(B)** Users' corresponding social workers can know their clients' current situation by using the web page; **(C)** All data will be stored in a secure server and double backuped, only doctors and executives are eligible for access.

The main structure of the system consists of three parts: (a) an APP for users; (b) a web page for service providers such as social workers; (c) server to store data and information (see Figure 1).

**Figure 2:**
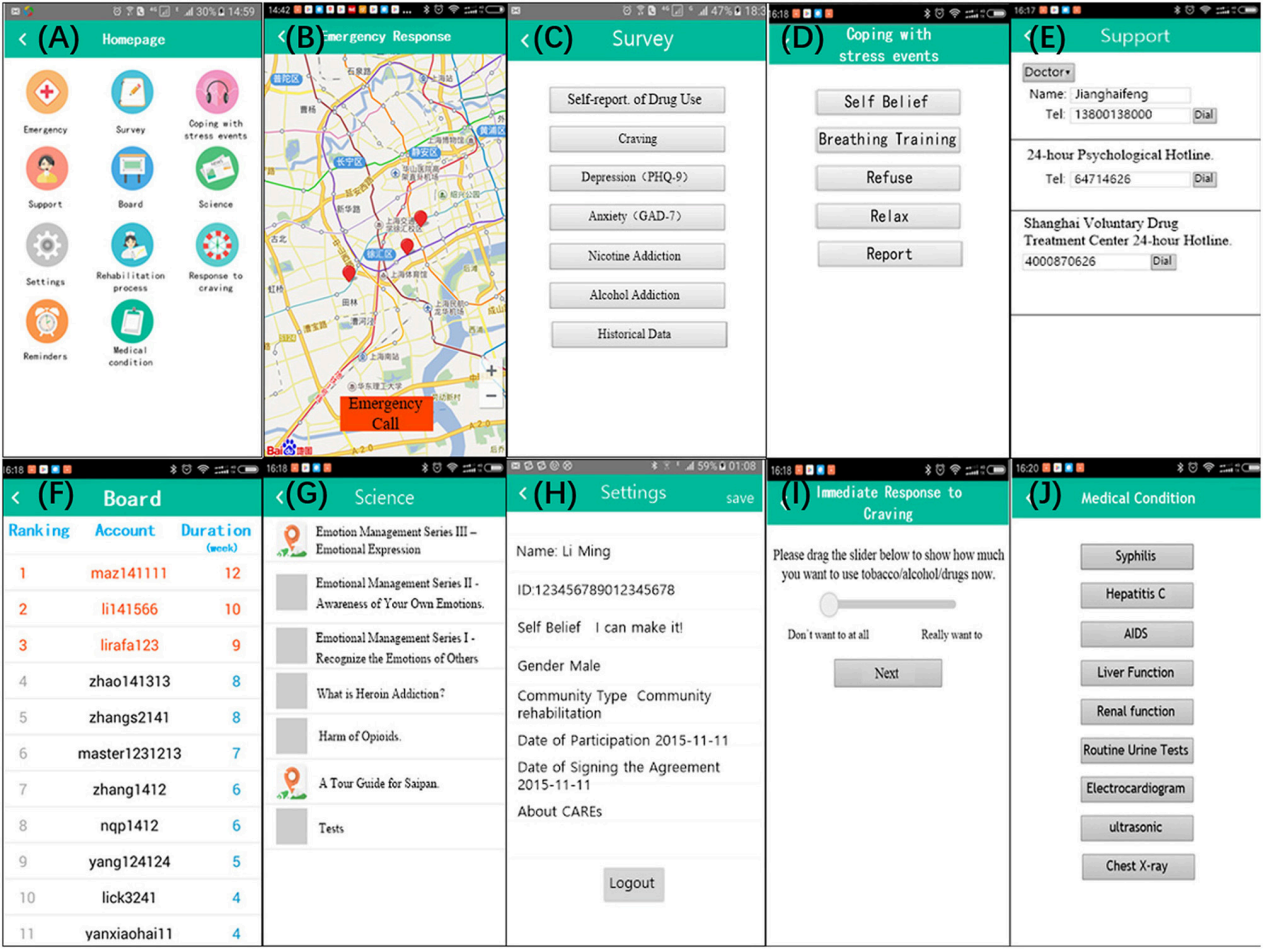
“Screenshots” taken on a phone device illustrating the user interface: no Regular reminders and Rehabilitation Process Management above for: (1) Regular reminders will only be used when users receive messages from service providers or server; (2) Rehabilitation process involves in some privacy of the patients. For better understanding, we translated some Chinese on the pages into English. CAREs actually is presented in Chinese.

The app provides disease and health knowledge, education, screening, assessment and monitoring, craving and emergency coping skills, and patient self-management to improve recovery outcomes among drug users in communities (see Table 1 and Figure 2).

**Table 1:** Corresponding function of each component of the mobile terminal of the community-based addiction rehabilitation electronic system(CAREs).

**Component**	**Function**
Setting and personal information	If the users use the addiction substance except cigarette and alcohol, then they need to add real name, user name (voice-print password), the main use of drugs, community drug addiction/rehabilitation information
Regular reminders	People who are enrolled in the community drug addiction/rehabilitation will be reminded to do the urine test (Community addiction: the first year once a month, the second year once every 2 months, the third year once every 3 months; community rehabilitation: the first year once every 2 months) Drug abusers has left the community addiction/rehabilitation, etc
Survey (every week)	Periodically evaluated and presented curves. Assessment information include: drug use in the past week, craving (visual simulation, VAS), depression (PHQ-9), anxiety (GAD-7), nicotine dependence scale (FTND)
Popular science pushing	According to types of the material and ASSIST score which was checked by users, the content of science popularization of drug rehabilitation and propaganda was pushed
Immediate response to craving	Craving assessment (using visual simulation, VAS), the coping methods for craving, include guided language, relaxation training (music, video, etc.)
Rehabilitation process management	Combine the information collected by Survey, pushing intervention based on time
Coping with stress events	Stress/Trigger assessment (time, location, nature of events), targeted push intervention
Board	According to Survey, the network is made up of the users and the top of information was showed
Support and outreach	Transfer of external resources, providing professional manual service hotline. For example, the 24 h hotline for the addiction department of Shanghai mental health center and the 24 h hotline for social workers
Emergency response	Push the first aid common sense, locate the nearby emergency hospital, and so on for emergency use
Medical condition	urinary drug testing (different types), syphilis qualitative, hepatitis C qualitative, HIV qualitative screening, urinary routine, liver function(ALT, AST),renal function (urea nitrogen, creatinine), electrocardiogram, B-ultrasonography, thoracic ultrasonography (X-ray).

**Figure 3:**
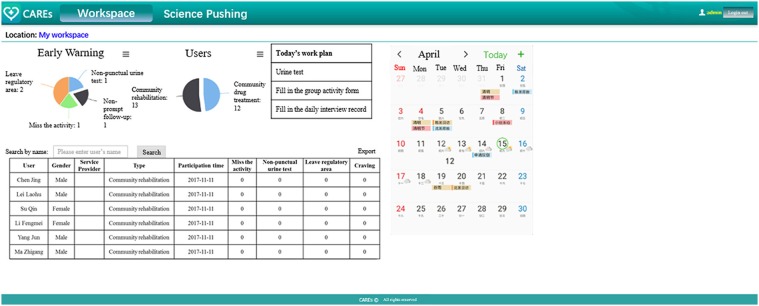
The main “screen” of the service provider web portal. For better understanding, we translated some Chinese on the page into English. The webpage is actually presented in Chinese.

The corresponding social workers can be linked with their clients via CAREs webpage. By using the webpage, social workers could review their corresponding clients' survey (self-report craving, stress events, medical laboratory testing results, and location information) and could give a response or arrange a face-to-face meeting accordingly. Social workers will also receive reminds and messages automatically, so that they could know who misses urine test, or the time who reports high craving or stress events (see Table 2 and Figure 3).

**Table 2:** Corresponding function of each component of the service provider terminal of the community-based addiction rehabilitation electronic system(CAREs).

**Component**	**Function**
Login/register	Service information
Group management	To achieve docking and management between the service and mobile terminal users
Information summary	Information include: The number of times users use the mobile terminal, rehabilitation process information (in time for the horizontal axis), Survey information (craving, stress events, medical laboratory testing), location information
Reminder	According to the situation of each mobile terminal user, push urine test reminder, treatment reminder, high-risk situation reminder (mobile terminal users report high desire or stress events)
Interaction	Push information (implemented on the “support and outreach” section of the mobile terminal).

Data including account information, statistics of user end, mobile user location information, and interaction between mobile users and social workers will be stored on a secure server. Registering to use CAREs is free and participants will receive an extra 50 RMB as compensation of potential cost on mobile data during the study period. At the end of the study, participants in integrated rehabilitation group could have the chance to gain the smartphone as a motivation to comply with the research protocol.

### Outcomes

The primary outcome is the performance on urine drug screen (UDS) which will be carried out regularly during the study period twice per week. The results of urine tests will be examined in the difference of proportion of drug-positive samples between two groups. Urine test kit will be used to test drug use including heroin, amphetamine-type stimulants, marijuana, cocaine, and ketamine. A subject who does not submit urine samples during the intervention period or who refuses to submit a sample even if he/she is present at the site will be considered as having a positive UDS.

Secondary outcomes include: (a) longest duration of sustained abstinence, defined as the longest continuous record weeks of negative urine drug screen samples; (b) interaction with social workers in the addiction recovery services: the days that participants interact with their corresponding social workers will be collected during the study period. Any phone call, SMS, face-to-face meeting, or communication via CAREs will be considered as one interaction; (c) the decrease rate of addiction-related issues severity index: the Chinese translation of the Addiction Severity Index (ASI) that has good reliability and validity in China will be used to assess the severity of addiction at enrollment and 6 months after enrollment. The reliability and validity of the Chinese version of the instrument have been assessed (21, 22). The internal consistency of the seven dimensions was judged to be acceptable (alpha = 0.44~0.79), the test-retest reliability was good (ICC = 0.68~0.84), and the inter-rater reliability was good (ICC = 0.87~0.98). The descent rate will be counted via dividing the minus value between baseline score and 6 months score by baseline score (assessments completed at assessment phases shown in Table 3).

**Table 3:** Assessments completed at assessment phases.

**Assessments**		**Assessment Phase**		
		**T1**	**T2**	**T3**
**BASELINE**				
	Drug use and UDS	X		
**PRIMARY OUTCOME**				
	The performance on UDS		X	X
**SECONDARY OUTCOMES**				
	Longest duration of sustained abstinence			X
	Interaction with social workers			X
	The decrease rate of addiction-related issues severity index			X

*T1: Baseline assessment*.

*T2: Duration of 6-month intervention*.

*T3: Completion of 6-month intervention*.

### Participant timeline

**Figure 4:**
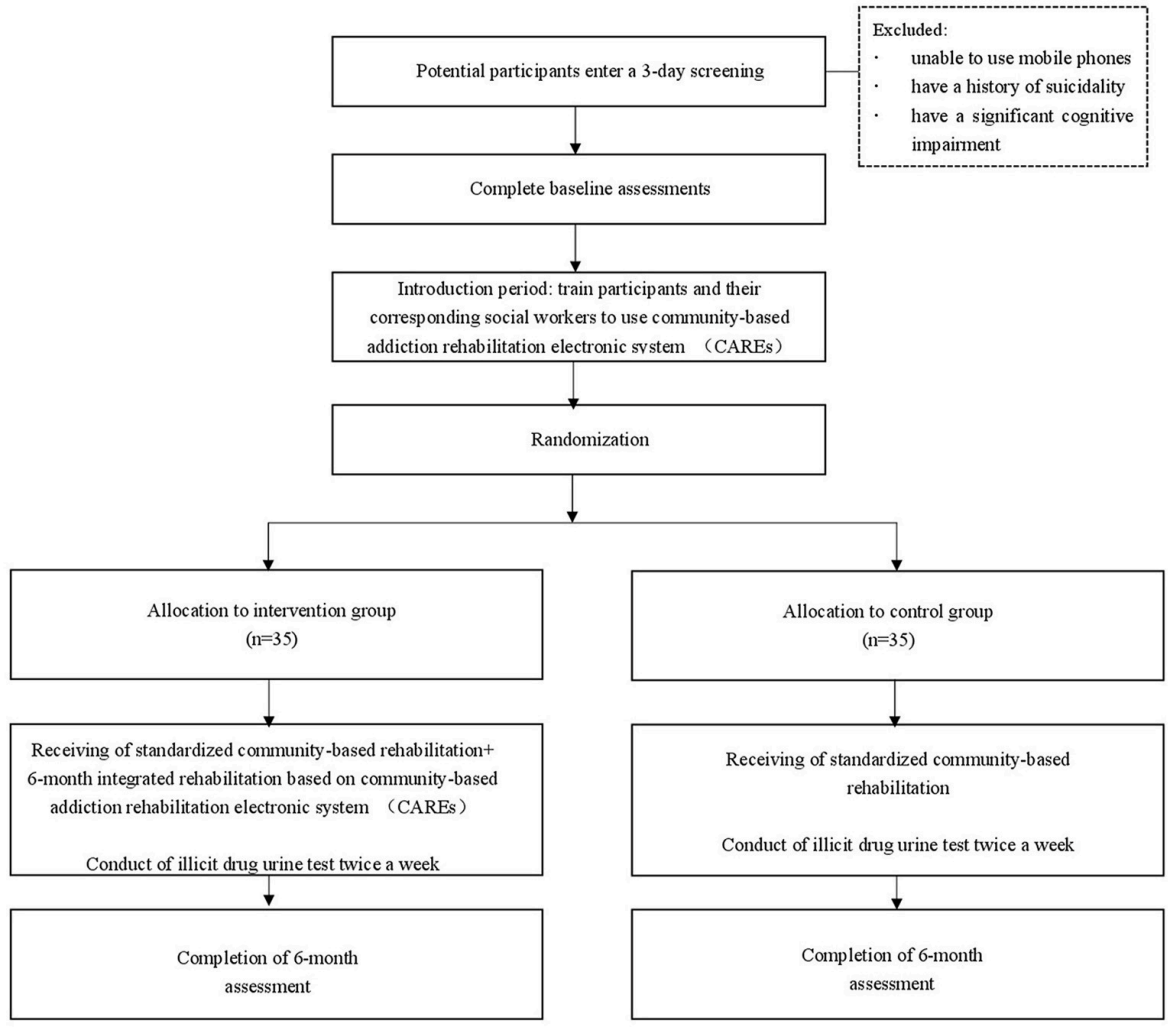
Participant flow diagrams.

Individuals who express interest will be invited to participate in informed consent procedures. Those who voluntarily consent to participate will then enter a 3 days screening to determine whether they meet inclusion/exclusion eligibility criteria. Baseline measurements of drug use and UDS will also be collected at this time. Participants who continue to meet eligibility criteria will participate in a 7 days induction period. During the induction period, participants and their corresponding social workers will be trained by technicians to get familiar with CAREs. Participants will be randomly assigned, in a 1:1 ratio, to receive either the intervention program or control program for a period of 6 months. Subsequent assessment points coincide for both groups at 6 months after randomization (see Figure 4).

### Discontinuation

The end of the study is defined as participants finishing the evaluation at the end of 6 months, or withdrawal from the study. Subjects are free to withdraw from the study at any time for any reason. They could also be withdrawn by the investigator, if necessary, to protect their health and the safety or integrity of the study data. The principal investigator could withdraw a participant from the study for any of the following reasons: (a) rehabilitation failure (having incarceration or readmission to compulsory treatment or both; (b) a protocol deviation that might compromise data integrity, protocol compliance or subject safety; (c) a participant's request to be discontinued from the study (i.e., a subject declines further study participation).

### Sample size

This is a pilot study design. Based on official records of the community-based rehabilitation program in Shanghai (23), the investigators estimated that the proportion of drug abusers in this program is around 40% and the proportion of loss-to-follow-up is 20%. Related literature pointed out that the use of mobile APP can result in a 60% reduction in substance use. Assuming α = 0.05, β = 0.10, a 70 participants sample size was calculated by sample size estimation for a comparison of two proportions. The sample size required for comparing the difference of performance on illicit drug urine test in two group (controlled group vs. integrated rehabilitation group) is 35 cases per group.

### Recruitment

Study participants will be recruited at social workers station in Shanghai. Drug abusers who are newly ordered to undergo community-based rehabilitation will be invited. Social workers who are trained will be responsible to provide consent and complete the screening tool. Recruitment has commenced and will continue until the end of 2018 or until our sample target is reached.

### Assignment of interventions

#### Allocation

Those that consent will be randomized using simple randomization tables generated by SPSS Statistics (version 22) (24); 35 subjects will be assigned to the usual group (routine community-based rehabilitation) and 35 to the integrated rehabilitation group (routine community-based rehabilitation + CAREs intervention).

#### Blinding

To minimize the risk of biased results as much as possible, the persons who conduct the urine tests and complete the evaluation of the ASI will be different from those who provide the intervention. But subjects may talk about the CAREs intervention during the assessment so it is not possible to completely “blind” the evaluators to the integrated rehabilitation group of the subjects.

### Data collection

The majority of study-specific data outlined in the protocol will be entered onto the paper Case Report Form (CRF) by site coordinators in accordance with the Clinical Completion Guidelines. Urine test data will be collected by each participant's corresponding social worker. These paper gees will be collected at each monitoring visit and double data will be entered by specialists. The CAREs usage data among integrated rehabilitation group will be collected automatically from the system, whereas those electronically data will be maintained on a secure server at SMHC and will be downloaded periodically for storage in a password-protected data file accessible by two study personnel.

### Data management

Intention-to-treat principle will be used in the statistical analysis. All statistical analyses will be conducted using SPSS Statistics (version 22) (24). Chi-square tests and ANOVAs will be used to compare characteristics of the members of the two groups. Primary outcome and secondary outcomes (results of urine illicit drug tests between the groups, the days that participants interact with their corresponding social workers) will be compared between groups using analysis of ANOVAs. Considering behavioral clinical trials often find data missing at the end of trials due to incomplete evaluations, Generalized Estimating Equations (GEE) will be used to compare the seven dimensions scores of the ASI between group across the study period. Any unfavorable changes to participants during the study period will be recorded. Since this study does not impact participants' routine care and is examining the effect of an integrated community-based rehabilitation for drug abusers in communities, no serious adverse events are anticipated and no interim analyses are planned.

### Monitoring

The monitoring authority at the Institute of Clinical Trial in SMHC will be utilized for good clinical practice (GCP) monitoring service during the study. Monitoring will occur from the time the first patient enters the study and continue until the last patient completes or discontinues participation via periodic visits. A clinical event committee (CEC) consisting of 3 expert members are responsible for differentiating diagnosis and/or adjudicating the final diagnosis during clinical trials based on protocol definition, to allow more accurate assessment of the test results. All CEC members are independent of the investigators; they are not directly involved in the trial and have no conflict of interest potentially affecting their impartiality and independent decision-making.

### Ancillary and post-trial care

At the end of the study, participants from integrated rehabilitation group have the chance to gain the smart-phone installed with the study APP as a gift accordingly. All participants will be maintained in community-based rehabilitation after this study.

### Research ethics approval

#### Institutional review boards

The Ethical Board of SMHC approved the study protocol (2017–33). Any significant modifications to the protocol will be forwarded to the committee for approval. This study is conducted in accordance with the Declaration of Helsinki of the World Medical Association (Seventh Revision, 2013). It complies with the International Conference on Harmonization Good Clinical Practice (ICH-GCP) guidelines and applicable Chinese regulatory requirements.

#### Consent or assent

Study candidates will present for the informed consent process and eligibility screening. The nature, purpose, potential risks and benefits, and requirements of the study will be explained to all candidates by trained researchers, with ample time for them to ask questions about the study. The nature and purpose of the informed consent process will also be explained to study candidates. After sufficient time to consider participation, those who wish to continue will be asked to sign and date the Informed Consent Form (ICF). The principal investigator or designee obtaining informed consent will also sign the ICF. A copy of the signed ICF will be given to every participant.

#### Confidentiality

All participant-identifying documentation generated in this study will be considered confidential and will not be disclosed to any persons not directly concerned with the study without written permission from the subject. However, authorized public security officials (or their representatives) will be allowed full access to inspect and copy the records.

#### Dissemination

After study completion, the results will be punished in international peer-reviewed journals. If the electronic system CAREs shown to be helpful in improving recovery outcomes in drug abusers in the community, it will be available for public to download.

## Results

We recruit research objects in communities in Shanghai with the aim of recruiting 70 subjects. Recruitment will continue until the end of 2018 or until our sample target is reached.

## Discussion

The purpose of this study is to provide health education, disease screening, assessment and monitoring, immediate support and intervention, and patient self-management for persons with substance use disorders.

Experimental Group and the matched social workers will accept the routine community drug addiction/rehabilitation treatment and a common use “CAREs” system. At the end of the 6 months, follow-up will be performed on the intervention group, including the completion of the community's addiction/rehabilitation content, the regular urine test negative rate. The validity of the “CAREs” system will be determined by comparing the results of the experimental group with the control group. It is anticipated that the intervention group will have less negative urine drug test samples and more interaction with social workers.

The project uses mobile medical technology as a foothold to explore the feasibility and effectiveness of using the mobile phone APP in the process of community drug addiction/rehabilitation treatment. The experience of this research will not only provide theoretical basis and model demonstration for clinical practice, but also has important significance for improving the ability of rehabilitation for substance addiction in China. It should be noted that this study is only conducted in Shanghai, so caution should be exercised when extending the results to other parts of China.

## Author contributions

ZW and SC are co-first authors and contributed equally to this work. JC and CX are responsible for system development and technical support. The other authors are team members of the program and have made a lot of contributions in trial design and thesis writing.

### Conflict of interest statement

JC and XC are employed by company Nantong Winner Information Technology Co., Ltd. The remaining authors declare that the research was conducted in the absence of any commercial or financial relationships that could be construed as a potential conflict of interest.

## Abbreviations

mHealth: mobile health
RCT: randomized controlled trial
CAREs: community-based addiction rehabilitation electronic system
DSM-5: Diagnostic and Statistical Manual of Mental Disorders-V
SMHC: Shanghai Mental Health Center
UDS: urine drug screen
ASI: the Addiction Severity Index
CRF: Case Report Form
GEE: Generalized Estimating Equations
GCP: good clinical practice
CEC: clinical event committee
ICH-GCP: International Conference on Harmonization
ICF: Informed Consent Form in China.
